# Pulsatile microvascular cerebral blood flow waveforms change with intracranial compliance and age

**DOI:** 10.1117/1.NPh.11.1.015003

**Published:** 2024-01-20

**Authors:** Nikita Kedia, Michael M. McDowell, Jason Yang, Jingyi Wu, Robert M. Friedlander, Jana M. Kainerstorfer

**Affiliations:** a University of Pittsburgh School of Medicine, Department of Neurological Surgery, Pittsburgh, Pennsylvania, United States; b Carnegie Mellon University, Department of Biomedical Engineering, Pittsburgh, Pennsylvania, United States

**Keywords:** aging, biomarkers, cerebral blood flow, cerebral hemodynamics, cerebrospinal fluid

## Abstract

**Significance:**

Diffuse correlation spectroscopy (DCS) is an optical method to measure relative changes in cerebral blood flow (rCBF) in the microvasculature. Each heartbeat generates a pulsatile signal with distinct morphological features that we hypothesized to be related to intracranial compliance (ICC).

**Aim:**

We aim to study how three features of the pulsatile rCBF waveforms: the augmentation index (AIx), the pulsatility index, and the area under the curve, change with respect to ICC. We describe ICC as a combination of vascular compliance and extravascular compliance.

**Approach:**

Since patients with Chiari malformations (CM) (n=30) have been shown to have altered extravascular compliance, we compare the morphology of rCBF waveforms in CM patients with age-matched healthy control (n=30).

**Results:**

AIx measured in the supine position was significantly less in patients with CM compared to healthy controls (p<0.05). Since physiologic aging also leads to changes in vessel stiffness and intravascular compliance, we evaluate how the rCBF waveform changes with respect to age and find that the AIx feature was strongly correlated with age ($R_{\text{healthy subjects}}=-0.63$, $R_{\text{preoperative}\;\mathrm{CM}\;\text{patient}}=-0.70$, and $R_{\text{postoperative}\;\mathrm{CM}\;\text{patients}}=-0.62$, p<0.01).

**Conclusions:**

These results suggest that the AIx measured in the cerebral microvasculature using DCS may be correlated to changes in ICC.

## 1 Introduction

Noninvasive bedside compatible tools to monitor cerebral health could greatly impact clinical care by reducing the need for invasive procedures. Diffuse transcutaneous optical modalities are becoming increasingly popular tools to monitor cerebral health. One such modality, diffuse correlation spectroscopy (DCS) uses the temporal fluctuations of scattered near-infrared light caused by moving red blood cells to monitor relative changes in cerebral blood flow (rCBF) in the microvasculature. These devices are noninvasive and easy to use, making them an attractive option for clinical applications.^1^^–^^4^ The use of DCS in clinical settings has been explored for various applications, such as predicting intracranial pressure (ICP),^5^^–^^7^ measuring dynamic cerebral autoregulation in stroke patients,^8^ monitoring intraventricular hemorrhage vulnerability in newborns,^9^ and estimating critical closing pressure (CrCP).^10^^,^^11^

Currently, transcranial doppler ultrasound (TCD) is the gold-standard method to measure the cerebral blood flow velocity (CBFV) in the large arteries of the brain. With CBFV, it is possible to generate pulsatile waveforms, which are the result of pressure and volume changes driven by the cardiac cycle. In healthy individuals, the shape of the pulsatile CBFV waveforms is characterized to be triphasic such that the first peak (P1) is related to the systolic flow, the second peak (P2) is related to the blood pulse wave reflections from closing heart valves along with the Windkessel effect, and the third peak (P3) is associated with the diastolic blood flow.^12^ There have been studies that characterize the morphology of CBFV waveforms and show that shapes of these waveforms are influenced by factors such as vascular stiffness and ICP.^13^ However, DCS has several advantages over TCD since it is less operator-dependent and does not require the need of a temporal bone window, which some patients lack.^14^ Due to the high sampling frequency in recent DCS systems, extraction of pulsatile rCBF waveforms, similar to the CBFV waveforms measured with TCD is possible. Unlike TCD, DCS measures rCBF at the level of parenchymal cerebral microvasculature, which has distinct regulatory properties compared to the large arteries within the subarachnoid space that TCD measures from.

Recent work has investigated using pulsatile rCBF waveforms measured with DCS as potential biomarkers for ICP and CrCP in clinical settings.^5^^–^^7^^,^^10^^,^^11^ However, there are limited studies quantifying what factors impact the morphological features of DCS pulsatile waveforms.^15^ It is believed that the rCBF waveforms measured have distinct characteristics from pulsatile blood flow waveforms in the periphery due to their confinement by a rigid skull. The Monro-Kellie doctrine describes that there are three compartments in the brain: the brain parenchyma, the cerebrospinal fluid (CSF), and the intracranial blood.^16^ The different compartments can act as a buffer such that if the volume of one of these compartments increases, the other two will decrease to control ICP under physiologic conditions.^16^ This implies that in the brain, compliance is not only reflective of how much the blood vessels can distend but is also mediated by extravascular compliance. The combined effects of the vessel compliance and this extravascular compliance is referred to as intracranial compliance (ICC).^13^ Several studies have shown that the shape of the CBFV waveform in the macrovasculature measured with TCD will change with increased ICP and with decreased ICC.^13^^,^^17^^,^^18^ Thus we hypothesize here that ICC also leads to morphological changes in the pulsatile rCBF waveforms measured with DCS.

Here we study the pulsatile blood flow waveforms in patients with Chiari malformations (CM) relative to healthy controls. CM is an anatomical malformation characterized by cerebellar tonsillar descent (TD) into the foramen magnum. This leads to disruptions in the free flow of CSF at the craniospinal junction that potentially alters the ability of CSF to shift to the intraspinal space in settings of elevated ICP.^19^^,^^20^ The classical, and most surgically correctable, symptom of CM is suboccipital headaches worsened by Valsalva maneuvers.^21^ Several studies have suggested that although patients with CM have a normal mean ICP value, the pulsatile ICP waveform, and the pulsatile CSF waveform change significantly in these patients due to decreases in ICC.^22^^–^^26^ Because CM is primarily an extravascular compliance issue with minimal effects on the cerebral vasculature, we investigate how ICC and pulsatile rCBF waveforms change in these patients compared to age-matched healthy controls and investigate what happens after surgical intervention. Further, we look at how the waveforms change with age since it is known that vessel stiffness increases, and vascular compliance decreases with age.^12^^,^^27^^–^^29^ Specifically, we assess the pulsatility index (PI), augmentation index (AIx), and the area under the curve (AUC) of the waveforms. These are features that have been studied in the TCD field. PI is defined as the difference between the peak flow value and the lowest flow value divided by the mean flow and has been shown to be related to arterial compliance and ICP.^30^^,^^31^ AIx is defined as the ratio of P1 to the P2 that captures the reflected wave augmentation and has been shown to correlate with vascular stiffness.^12^^,^^32^ Finally, the AUC has been shown to be an important feature when predicting ICP noninvasively with pulsatile DCS waveforms.^5^^,^^6^ Here we examine how these three features change in patients with CM and with vascular stiffness driven by normal aging.

## 2 Methods

### 2.1 Participants

Type 1 CM patients (n=32) were enrolled in this study during their preoperative appointment in an outpatient Neurological Surgery Clinic at the University of Pittsburgh School of Medicine Presbyterian Hospital, Pittsburgh, Pennsylvania, United States. Diagnosis of symptomatic type 1 CM was made based on evidence of TD on magnetic resonance imaging (MRI) and clinical symptoms. TD and CSF obstruction were determined by a board-certified neurosurgeon using MRI.

Out of 32 patient recordings, 2 recordings were not included due to technical issues with the recording. Repeat postoperative recordings were performed on n=27 subjects. Initial DCS recording occurred an average of 9±13 days before surgery. Follow-up recording was performed at the postoperative appointment, which was on average 16±4 days after the surgery. All enrolled patients underwent Chiari decompression surgery by a single surgeon (R.F.), which included a standardized technique of suboccipital craniectomy, C1 laminectomy (partial or complete), and expansile duraplasty. This surgery serves to increase the space in the posterior fossa allowing for improved CSF flow across the craniocervical junction.

Age-matched healthy volunteers (n=32) were enrolled at Carnegie Mellon University and in a spine clinic at UPMC. Exclusion criteria for the healthy controls included history of high blood pressure (BP) (>150/95), diabetes, history of blood clots, blood thinners, or pacemaker. Two healthy subjects were excluded because they were not able to complete the experiment either due to time constraints or discomfort of the DCS probe on the head. The study protocol was approved by both the University of Pittsburgh and Carnegie Mellon University Institutional Review Boards. Written informed consent was obtained from healthy controls and CM patients.

### 2.2 Diffuse Correlation Spectroscopy

In this study, we used DCS to extract pulsatile blood flow cardiac waveforms. DCS is a diffuse-optical modality that uses near-infrared light to monitor cerebral blood flow in the microvasculature. More information about the principles of DCS can be found elsewhere.^4^^,^^33^^,^^34^ Briefly, the decay time of the temporal autocorrelation of a speckle pattern generated from a long coherence-length laser is converted into the electric field autocorrelation. This is fitted to a semi-infinite analytical model to measure variations in blood flow. The rCBF can be measured as $\alpha D_{b}$, where α is the fraction of photon scattering events that occur due to red blood cells in the tissue, and $D_{b}$ is the motion of red blood cells modeled through Brownian motion.

Here we used a similar instrumentation as described in the previous studies.^5^^,^^35^ A custom-built DCS with an 852 nm (TOPTICA Photonics AG, Germany) long-coherence-length source and four single-photon counting modules (Excelitas Technologies Corp., Canada) were used. We used a flexible rubber probe with a source–detector (SD) distance of 2 cm (Fiberoptics Systems Inc., United States). The probe was secured on the forehead with double sided medical tape along with a soft headband. This headband ensured that there was enough pressure present to minimize scalp blood flow. The DCS device had a 50 Hz temporal bandwidth and was oversampled and recorded at a 500 Hz sampling frequency. An image of the system and the probe can be seen in Fig. S5 in the Supplementary Material. Manual digital markers were sent to the auxiliary port of the DCS to indicate when BP was measured and to mark measurement protocol events.

### 2.3 Measurement Protocol

The DCS probe was placed on one hemisphere of the forehead corresponding to the anterior fossa contents, chosen based on patient comfort, ∼1 in. above the eyebrow. Participants were asked to rest in the supine position for 9 min, in the sitting position for 9 min and then were instructed to lean forward with their knees raised above their chest for 4 min in order to simulate a Valsalva maneuver. However, depending on the participant’s comfort, actual time for each posture varied. For CM patients, postop instructions strictly prohibited prolonged Valsalva maneuvers; therefore, no data were collected in the leaning forward position for the postop group. Further, two patients had pain around the incision site postoperatively and were not able to lay supine, thus only data in the sitting position was taken for these patients. The average time in each position is reported in Table 1. At the end of each posture, an automatic BP monitor (Welch Allyn, United States) was used to record BP. For 6 CM patients, a different BP cuff was used that measures BP at the wrist and has high rates of error, thus these data were excluded from the analysis.

**Table 1:** Number of participants in the first row indicates how many subjects were included in this study for each cohort: healthy subjects, preoperative CM patients, and postoperative CM patients.

	CM patients		
	Preoperative	Postoperative	Healthy
Number of participants included	n=30	n=27	n=30
Supine (time in min)	8.06 ± 2.01 (n=30)	7.99 ± 2.01 (n=25)	8.86 ± 1.31 (n=30)
Sitting (time in min)	8.02 ± 1.62 (n=30)	8.04 ± 1.59 (n=26)	8.86 ± 0.84 (n=30)
Valsalva (time in min)	3.41 ± 1.15 (n=28)		3.92 ± 0.81 (n=29)
Age (years)	35.1 ± 9.8		34.4 ± 11.5
Sex, female (%)	87.5		62.5
Race, White (Non-Hispanic) (%)	96.9		59.4
Mean arterial pressure (mmHg)	94.3 ± 7.8 (n=24)	93.7 ± 7.3 (n=21)	89.2 ± 9.2
HR (BPM)	69.8 ± 11.7	78.1 ± 14.0	69.4 ± 10.9
TD (mm)	9.55 ± 4.12		
Percent CSF at foramen magnum	18.9 ± 9.3%		
BMI	31.7 ± 6.1		

Note: The time in each posture and the number of participants who were able to complete a given posture varied. Continuous variables are reported as mean (standard deviation) and categorical variables are represented as percentages. MAP and HR were calculated from the supine position for each cohort. For the CM patients, average BMI, TD, and percent CSF were also calculated.

In some cases, certain postures were too painful for patients or there was too much movement leading to noisy signals where the pulsatile features of the rCBF waveforms could not be extracted. In these cases, only a subset of the patient or subject’s data was used. The total number of patients included for each posture is indicated in Table 1. Additionally, demographic information (age, sex, and race) is presented for the healthy controls and CM patients. The body mass index (BMI) of the CM patients is also reported and determined through retrospective medical chart review. BMI was not collected in the healthy subjects.

### 2.4 Signal Processing

All signal processing was performed using MATLAB R2022b (The MathWorks Inc., Natick, Massachusetts, United States). CBF was normalized for each recording by dividing $\alpha D_{b}$ by its average over the entire time series. Motion artifacts such as those associated with posture changes led to large spikes in the data, thus these were removed from the signal before calculating the average by first performing a z-score rejection (z>3) on the $\alpha D_{b}$ time series. The average obtained after z-score rejection was used to normalize $\alpha D_{b}$, and the normalized $\alpha D_{b}$ was used for the subsequent analysis, denoted as rCBF.

**Fig. 1:**
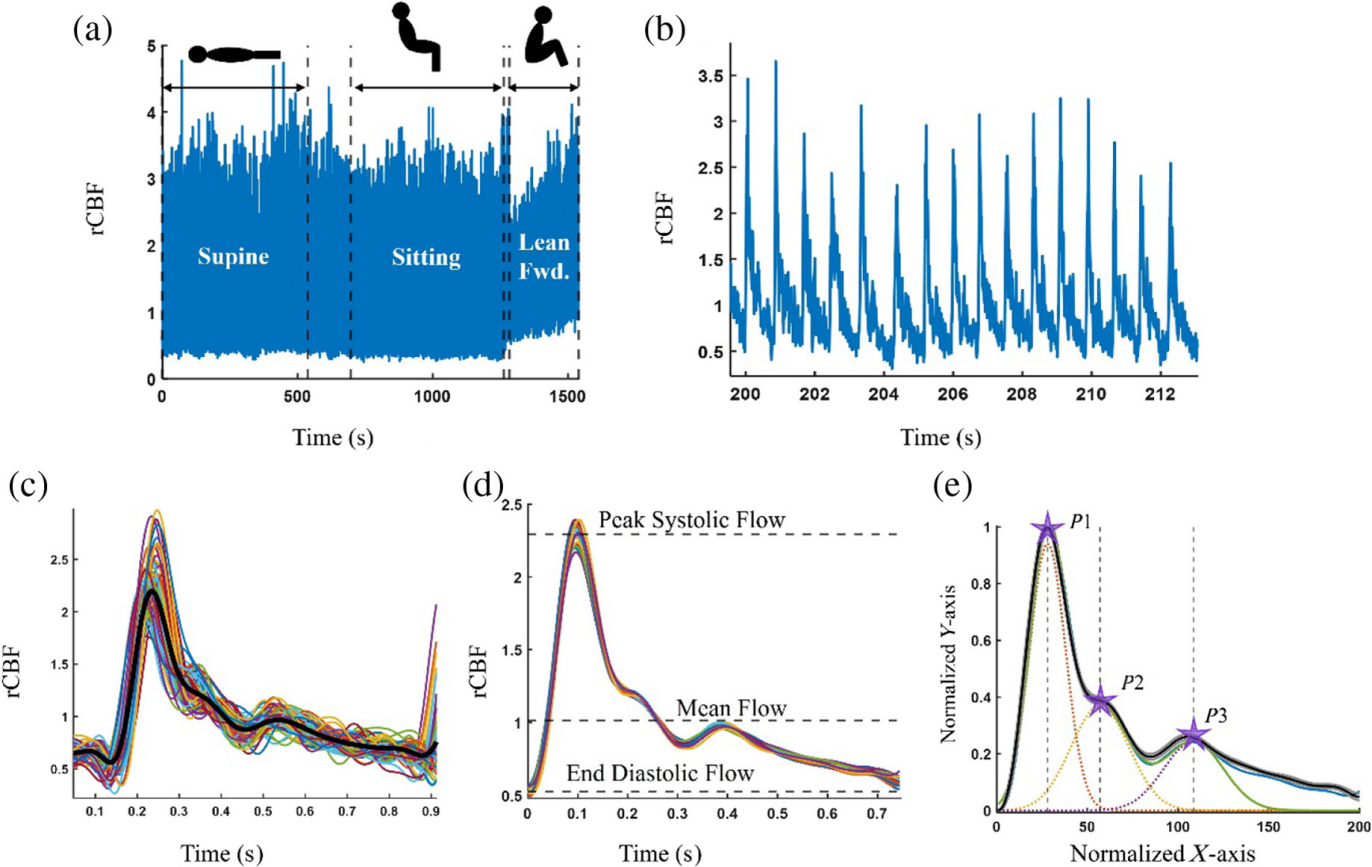
(a) DCS series from a recording in one healthy subject (F/23 years old) with dotted lines indicating when the markers were sent to the auxiliary port of the DCS to isolate each posture change. (b) A zoomed in region of 15 s of the DCS signal shows the pulsatile rCBF waveforms. We apply a BPF (0.3 to 10 Hz) and isolate individual pulses. (c) 78 individual rCBF pulses are shown corresponding to 1 min of recording along with the black line representing the average waveform generated over that one minute. For our analysis, we average pulses over a 1-min intervals with a moving window of 30 s. This leads to (d) 18 averaged pulsatile waveforms generated over the 9-min recording in the supine position. After normalizing the waveform, we fit three Gaussians plotted with dotted lines in (e) to the waveforms to identify the peaks.

The onset and end of each posture change were segmented out using markers sent to the auxiliary port of the DCS during the recording as seen with the dotted lines in Fig. 1(a). Periods of motion, such as when the participant was moving from one position to the next, were not included. Baseline changes in CBF (${\mathrm{rCBF}}_{0}$) for a given posture were calculated by first applying a low-pass filter (LPF) on the data using a Butterworth filter with a filter order of 20 and a cutoff frequency of 0.05 Hz. The ${\mathrm{rCBF}}_{0}$ was calculated by taking the average of the low-pass filtered data in the middle 1-min of a given posture. The LPF filter was only used to obtain ${\mathrm{rCBF}}_{0}$ and not applied when calculating the pulsatile features.

The pulsatile waveforms were isolated using a series of signal processing steps for each posture. First, a seventh-order Butterworth high-pass filter (HPF) with a cutoff frequency of 0.3 Hz was used to remove respiration. The heart rate (HR) frequency ($f_{\mathrm{HR}}$) was found using the fast Fourier transform. A seventh-order Butterworth LPF with a cutoff frequency 0.7 Hz higher than the extracted HR frequency ($f_{c}=f_{\mathrm{HR}}+0.7\;\mathrm{Hz}$) was used to find the period of an individual heartbeat. This narrow bandpass filter only retained the first harmonic of the HR and thus did not contain multiple peaks. The “findpeaks” function in MATLAB was used to find the location of each pulse. For the actual waveform shape extraction, a seventh-order Butterworth LPF with a cutoff frequency of 10 Hz was used after the HPF removed the respiration frequency. This was used to remove high-frequency noise from the signal while keeping at least four to five harmonics present in the signal. The rCBF curve was isolated by taking ∼0.5  s of data before and after each peak to isolate the entire pulse. However, this was not always consistently 0.5 s and the true range was based on the HR such that we could identify both troughs corresponding to the beginning and the end of a pulse. Following this, outlier pulses were removed if they had a z-score >3.

rCBF cardiac waveforms were averaged over 1-min to improve the signal-to-noise ratio shown in Fig. 1(c). Additionally, a moving window of 30 s was applied. The beginning and end of each averaged pulse were detected by finding the minimum values in the averaged waveform, corresponding to the beginning of systole and end of diastole. The pulsatile waveform was then normalized in the x and y directions before extracting pulsatile features to minimize variability heart rate. To normalize the waveform in the y axis, the minimum of the flow waveform was set to be 0 and the highest peak of the flow was set to be 1. The pulse was set to be 200 data points wide for all patients using the “interp1” function on MATLAB. This normalized waveform is referred to as ${\mathrm{rCBF}}_{\text{norm}}$. These steps were repeated for each of the postures (supine, sitting, and leaning forward).

### 2.5 Feature Extraction

Prior to normalization, we extracted the maximum flow, minimum flow, and mean flow of the averaged rCBF pulse [Fig. 1(d)]. The pulsatile rCBF waveforms are comprised of three separate peaks, as described previously.^6^ Here we used a semiautomated Gaussian fitting approach to estimate the location and height of each peak. Specifically, we fitted the sum of three Gaussian functions to each waveform.

The sum of the three Gaussians was expressed as

$$y=\Sigma_{i=1}^{3}(a_{i}/b_{i})\phi (k_{i}),$$

where $k_{i}=(x-c_{i})/b_{i}$. ϕ is the normal distribution’s probability density, x is the normalized x axis of the rCBF waveform, and $a_{i}$, $b_{i}$, and $c_{i}$ are the height, scale, and location of each Gaussian, respectively. We let each rCBF waveform be y^, and then fitted $a_{i}$, $b_{i}$, and $c_{i}$ by computing a mean-square-error loss L(y^,  y)=‖y^−y‖2 using “fmincon” in MATLAB. The range of these parameters was determined empirically by visually verifying the fit of the Gaussians to the rCBF waveform as seen in Fig. 1(e). The height $a_{i}$ was set to be between 10 and 50 for all three peaks, the range of the scale $b_{i}$ was set to be between 1 and 50 for all three peaks, and the range of the location $c_{i}$ was set to be between 1 to 80 for P1, 1 to 150 for P2, and 1 to 180 for P3.

The “fmincon” function on MATLAB starts with the initial values of $a_{0}$, $b_{0}$, and $c_{0}$ and attempts to find a minimizer of the loss function. The initial values of $a_{0}$ and $b_{0}$ were set be the same for all the waveforms (30 and 1, respectively). However, the values for $c_{0}$ were manually changed depending on roughly where the peaks were visualized. Here we were mainly concerned with fitting the first and second peak (P1 and P2). To optimize the performance of peak finding for these two peaks, we computed the mean-square-error loss on the first 60% of the rCBF waveform. Since, the x axis was normalized to 200 datapoints for each rCBF waveform, the loss function was defined as loss L(y^,y)=‖y^(1:120)−y(1:120)‖2. From this, we were able to extract the final values of the peak locations c calculated using “fmincon” for the location of the three peaks. The y values corresponding to these were used as the heights of P1, P2, and P3. An additional rejection criterion was added such that if the final value of the loss function was >0.2 that waveform was excluded from final averaging. A figure comparing this approach and traditional peak finding approaches, such as using “findpeaks” on MATLAB is shown in Fig. S1 in the Supplementary Material.

The three features extracted from the pulsatile waveforms included AIx, PI, and AUC, defined as follows:

$${\mathrm{AI}}_{x}=\frac{P1}{P2}\;\mathrm{PI}=\frac{\left(\text{maximum flow}-\text{minimum flow}\right)}{\text{mean flow}},\;\mathrm{AUC}=\sum {\mathrm{rCBF}}_{\text{norm}}.$$

### 2.6 Statistics

The Shapiro–Wilk test was performed to test for normality, but the null hypothesis was rejected suggesting a skewed distribution in our variables of interest (AIx, PI, and AUC). To account for this, we use nonparametric tests. Correlation was evaluated using the Spearman correlation. To compare differences between healthy and preop CM patients, we used the two-tailed Mann–Whitney U Test, and to compare differences between the preop and postop CM patients, we used the two-tailed Wilcoxon signed rank test. The preop and postop comparisons were performed between patients where we had full sets of data (seen in Table 1) since some patients could not tolerate certain postures. Additionally, we conducted an ANCOVA to compare AIx between preoperative CM patients and healthy controls, controlling for age as a covariate. The effect size in this analysis was quantified using Partial Eta Squared. To evaluate changes in the waveform based on posture, we only compared preop CM patients and healthy controls. We used a Friedman test to assess if there were statistically significant differences in systemic factors and between the rCBF waveform features for each of the postures. Mann–Whitney U was used to test differences between healthy subjects and CM patients. The alpha level was set to 0.05 for all analyses. Statistical tests were performed using the SciPy Package in python.

## 3 Results

**Fig. 2:**
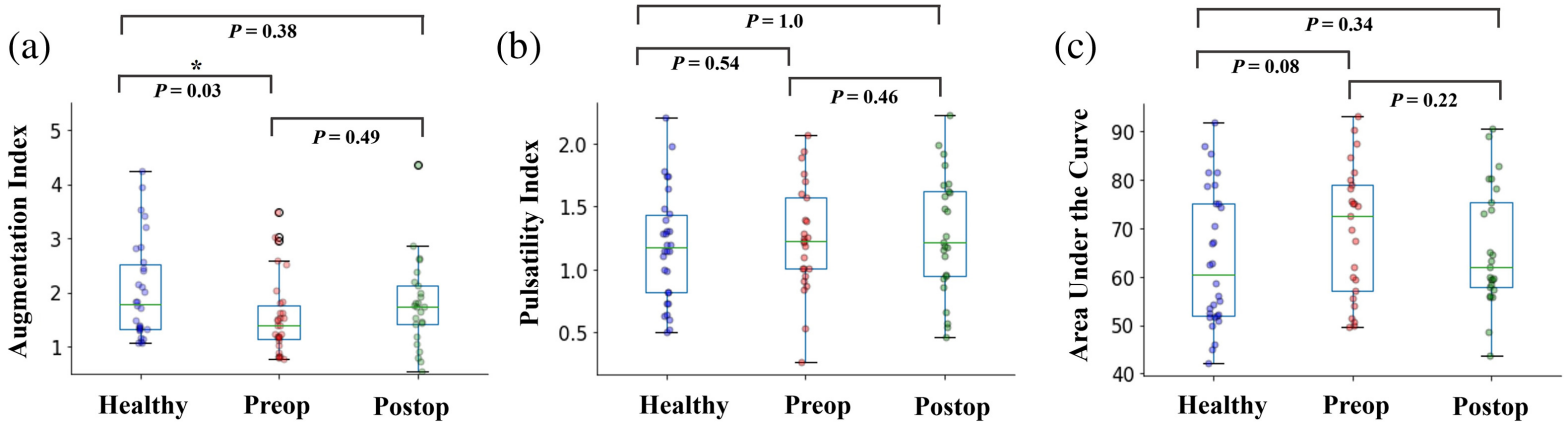
Box plots showing the difference between the pulsatile features [(a) AIx, (b) PI, and (c) AUC] calculated in the supine position in patients with CM and healthy controls. Mann–Whitney U tests are performed between the healthy subjects and the CM patients and paired Wilcoxon signed rank tests are performed between preop and postop CM patients. The p values are displayed above. The only statistically significant difference is between the AIx in healthy subjects and preop CM patients. Median values in the pulsatile features postoperatively increase or decrease in the same direction as the healthy subjects.

In Fig. 2, the supine measurements of AIx, PI, and AUC in healthy subjects were compared with results from CM patients both preoperatively and postoperatively in order to assess for changes in pulsatile features of the rCBF waveform. Healthy volunteers had a significantly higher AIx compared to CM patients preoperatively. Although the median AIx increased from preop to postop, this was not statistically significant as seen in Fig. 2(a). The PI was not significantly different in any of the groups as seen in Fig. 2(b). Additionally, the median AUC was lower in healthy subjects and after surgery in patients with CM, but these differences were not statistically significant as seen in Fig. 2(c).

**Fig. 3:**
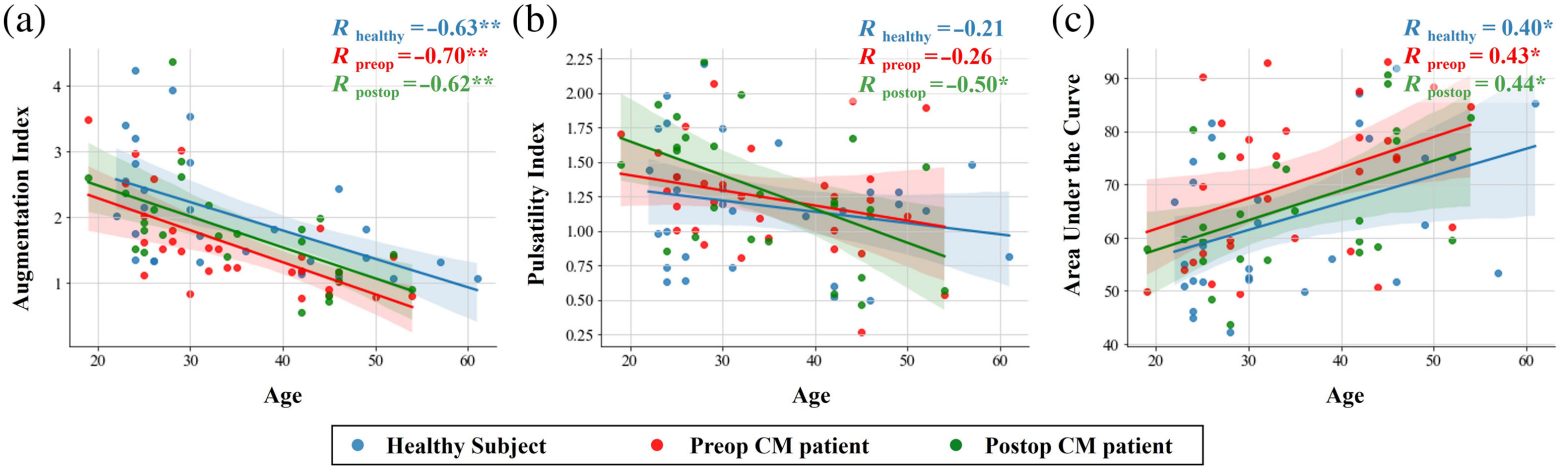
Correlation of the pulsatile rCBF waveform features, (a) AIx, (b) PI, and (c) AUC, with age in the three groups. Pulsatile waveforms are from when the subjects were in the supine position. Spearman correlation coefficients are displayed for each group, one star indicates p<0.05 and two stars indicate p<0.01.

In Fig. 3, we evaluated how age impacted the pulsatile features in the supine position. In Fig. 3(a), we see that AIx is highly correlated with age for all three groups (healthy subjects, preop CM, and postop CM patients). PI was weakly correlated with age; however, the correlation was highest for the postop CM group and was not significant in the preop CM group or in healthy subjects as seen in Fig. 3(b). Finally, in Fig. 3(c), we see that the AUC was significantly correlated with age for all three groups. Overall, AIx correlated most strongly with age. Results for the sitting position and leaning forward position are shown in Fig. S2 in the Supplementary Material, which also show a strong correlation between the pulsatile features and age.

Given that AIx was found to be correlated with age and was found to be statistically significant in the comparison between healthy patients and preoperative CM patients, we conducted an ANCOVA, incorporating age as a covariate. The regression model using age and type of subject (CM patient or healthy subject) as predictors of AIx accounted for ∼40.6% of the variance in AIx, evidenced by an R-squared of 0.406. The adjusted R-squared, which accounts for the number of predictors, was 0.385. Controlling for age, our analysis found that AIx in preoperative CM patients was, on average, 0.4552 units lower than in healthy controls, a difference that was statistically significant (p=0.01). The overall fit of the model was statistically significant as well, as evidenced by an F-statistic of 19.49 (p<0.001). Furthermore, a Partial Eta squared analysis was performed to look at effect sizes of CM and age on AIx. This analysis showed that the effect size of having CM was 0.1095 (p=0.01), whereas the effect size of age was more pronounced at 0.3518 (p<0.001). Thus age had a larger effect than the presence of a CM on AIx but both contribute significantly.

**Fig. 4:**
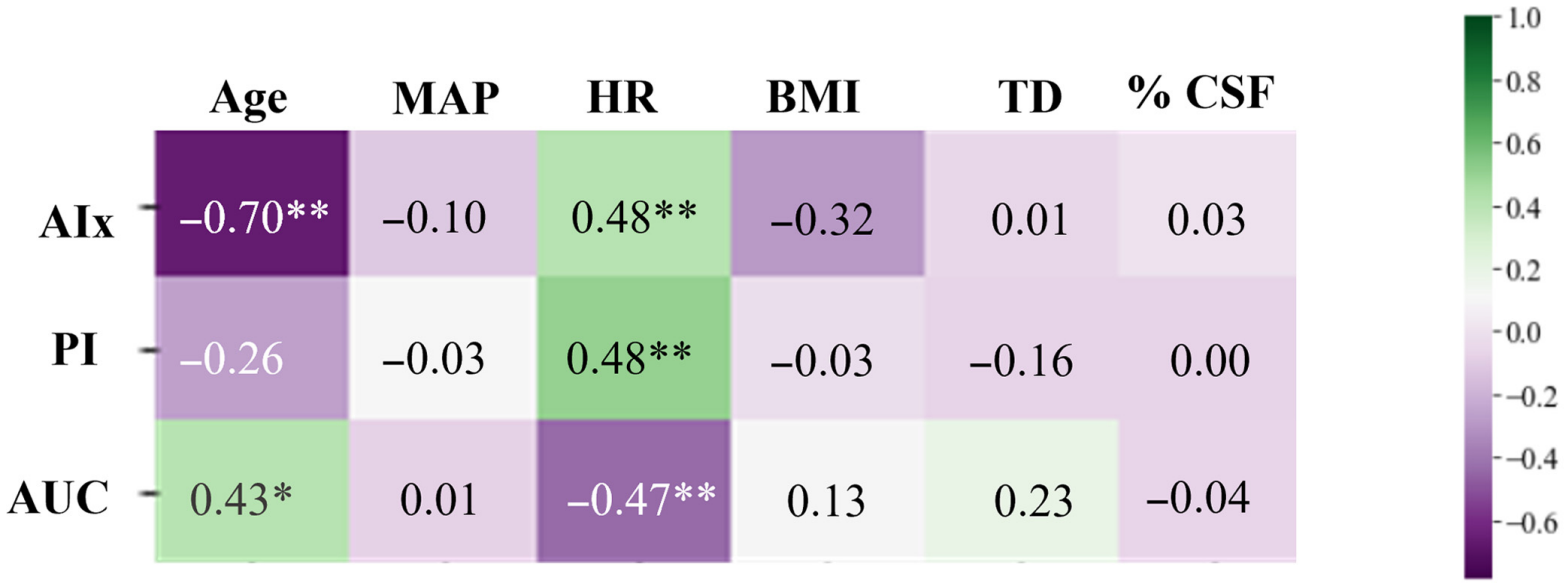
Heatmap showing the Spearman correlation of each of the pulsatile rCBF waveform features with age, MAP, HR, BMI, TD, and % CSF for preop CM patients in the supine position. One star indicates p<0.05 and two stars indicate p<0.01.

We also investigated how pulsatile rCBF features correlated with other patient specific factors in the preop CM patients. In Fig. 4, we show the Spearman correlation coefficient corresponding to each of the three rCBF features (AIx, PI, and AUC) with respect to age, MAP, HR, BMI, cerebellar TD, and the percent of CSF seen at the foramen magnum (based on MRI). For all three pulsatile rCBF waveform features, HR is significantly correlated to the features. As shown in Fig. 3, age was correlated with AIx and AUC but not PI for preop CM patients. The other factors (MAP, BMI, TD, and percent CSF) were not significantly correlated with the pulsatile features. Interestingly, in healthy subjects, we saw a higher correlation between the waveform features and MAP, but a lower correlation with respect to HR. Though, this was not statistically significant.

**Fig. 5:**
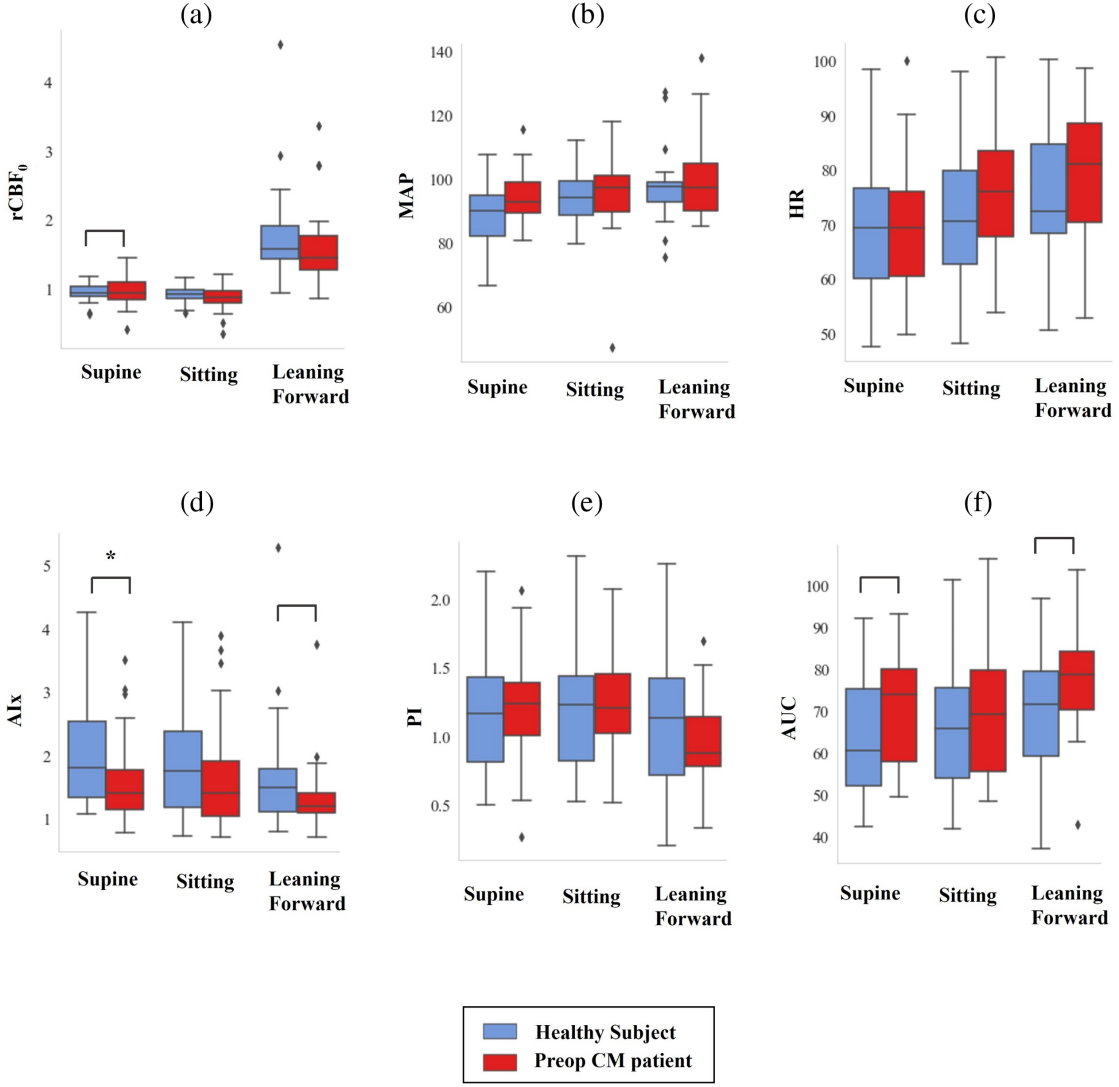
Changes in (a) ${\mathrm{rCBF}}_{0}$, (b) MAP, (c) HR, and the (d)–(f) pulsatile rCBF waveform features with various postures. The ${\mathrm{rCBF}}_{0}$, MAP, HR, AIx, and AUC were significantly different in the various postures in both preop CM patients and healthy subjects. PI was only significantly different with the postures for preop CM patients (and not healthy controls). Each variable was compared for preop CM patients compared to healthy controls. Empty brackets signify p<0.1 and stars signify p<0.05.

We also compared how systemic variables and pulsatile CBF waveform features change with respect to posture changes. Since patients were not able to lean forward postoperatively, we only consider preop CM patients and healthy controls. In Fig. 5, we evaluated how baseline CBF, MAP, and HR changed with posture for patients with CM compared to healthy controls. For both groups, healthy and preop CM patients, we saw statistically significant differences in the baseline CBF, MAP, and HR between the postures, determined using the Friedman test. Baseline CBF (${\mathrm{CBF}}_{0}$), MAP, and HR were not significantly (p<0.05) different between CM patients and healthy controls in any of the postures.

In Fig. 5, we also evaluated how the CBF waveform features (AIx, PI, and AUC) changed with respect to the posture changes. Using the Friedman test, we found that AIx and AUC changed significantly with changes in posture. PI was found to be significantly difference between postures in preop CM patients but was not significantly different in healthy subjects. The supine columns of the box plots of Fig. 5 are the same results as those shown in Fig. 2; however, here we also consider differences in the sitting and leaning forward position here. In all three postures, we see that the preop CM patients had a lower median AIx and AUC compared to the healthy subjects, however, only AIx in the supine position was statistically significant. PI was not found to be different between CM patients and healthy controls in any of the postures.

## 4 Discussion

In this work, we investigated how three features of the pulsatile CBF waveform (AIx, PI, and AUC) measured in the cerebral microvasculature change with ICC. We approximated decreased ICC by comparing patients with a CM to healthy controls and by evaluating age-related changes. We find that the AIx changed most significantly with these changes in ICC.

AIx is defined as the ratio of the first peak to the second peak in pulsatile waveforms. It has been well-documented that the AIx measured with other modalities is correlated with age-related changes in vascular compliance. This has been documented in many vascular beds including the radial artery,^36^ the aorta and carotid arteries,^32^ and the brain macrovasculature.^37^ There is also evidence that pulsatile waveforms change in the small arteries in the brain using MRI,^38^ but this has not yet been shown with DCS. There is some debate on whether AIx measured in these other modalities is truly correlated with vascular stiffness,^39^ but it is known that age is highly related to vascular stiffness.^40^ Here we see a very strong correlation with AIx measured with DCS and progressive patient age. This variation of AIx with age was strong in healthy controls and in Chiari patients, a total study population of n=60. We attribute these changes to changes in vascular compliance in the microvasculature.

However, we were also interested in how changes in extravascular compliance may impact the CBF waveforms. One study modeled the effect of extravascular compliance^13^ and provided evidence that the pulsatile waveforms measured with TCD are not only impacted by vascular stiffness, but in the enclosed skull, extravascular compliance leads to further changes in waveform morphology.^13^ It is also known that ICC is linked to ICP such that when ICC is low, a small change in volume will lead to a larger change in ICP.^41^ This is important to consider since several recent papers have related the pulsatile morphology of CBF waveforms,^5^^,^^6^ CBFV waveforms,^12^ and even cerebral blood volume pulses measured with NIRS^42^ to ICP. When using blood flow information to predict global ICP, it may be important to decouple whether changes in the pulsatile waveform are due to the vascular compliance or the extravascular compliance. Often increased ICP is not due to vascular changes but is instead driven by changes in the other compartments described by the Monro Kellie Doctrine.^16^ For example, in hydrocephalus there is excess CSF in the ventricles and in traumatic brain injuries there is brain tissue swelling. In these cases, changes in ICP are related to decreased extravascular compliance with little effect on intravascular compliance. Conversely, in certain cases of impaired autoregulation, such as some patients with severe traumatic brain injury, vascular compliance is the driving force in ICP perturbations and, thus, the treatment of ICP spikes is dramatically different than in the case of an extravascular compliance scenario.

We evaluate pulsatile CBF waveforms in CM patients to gain an understanding of the effects of extravascular compliance. CM has been shown to be related to decreased ICC using MRI studies and invasive ICP monitors.^19^^,^^21^^,^^22^^,^^25^ Since these invasive ICP monitors are not typically indicated for patients with CM, there are limited studies investigating how ICP changes in CM patients. However, some studies have shown that while the mean ICP is generally unaffected in patients with CM, the pulsatile ICP waveforms changes significantly.^43^^,^^44^ Thus CM impacts the ability for the brain to accommodate more volume leading to transient ICP spikes, but at baseline it does not lead to high ICP. This implies that we may be able to better isolate ICC, as opposed to ICP. Additionally, CM has not been shown to impact vascular compliance and is uniquely an isolated extravascular compliance issue.

It is also well-established that certain postures, such as Valsalva or leaning forward can exacerbate symptoms of CM, and there has been some evidence suggesting that the mean ICP changes more with postural changes in CM patients as compared to those without obstruction in the craniospinal junction.^45^ We saw significant differences in the pulsatile features with postural changes, but there were not strong differences between the CM patients and healthy controls in most of the CBF features. Other studies have also found that ICC markers change somewhat with posture.^46^ In our study, we saw that AIx decreased in the leaning forward position, which is shown to increase ICP and decrease ICC.^47^ Since symptoms get worse with leaning forward in patients with CM, we would have expected that there would be a more significant difference in the pulsatile features when patients are performing this posture, but we do not see this here. Reasons for this may include variability between patients in terms of how much they were able to lean forward, limited time in this position, and more focal changes in rCBF dynamics at the posterior fossa compared to the frontal lobe.

In this work, we assume that age-related changes are primarily due to vascular stiffness. This is not strictly true,^39^ and there is some evidence that extravascular compliance is also reduced in older adults though this was only seen when ICP was significantly elevated.^48^ Our results indicate that there are changes in the pulsatile CBF waveform measured with DCS in patients with CM even when compared to age-matched healthy controls. This finding may suggest that the decreased extravascular compliance in patients with CM is causing changes in the pulsatile waveform independent of the age-related changes that we see. Although we see significant differences between CM and healthy controls in AIx, the effect size of this change is much smaller than that driven by aging. We do not see any significant changes in the pulsatile features postoperatively, but on a group-level the AIx and AUC change in the direction of healthy controls (Fig. 2). However, there is a lot of variability in how the rCBF waveforms change postoperatively, which can be seen in Fig. S6 in the Supplementary Material, where we show supine rCBF waveforms pre- and postoperatively in three patients. These results may support the claim that changes in AIx are predominantly due to changes in cerebrovascular compliance with less effect of the extravascular compliance on the pulsatile waveforms. Additionally, the level of TD and the amount of CSF at the foramen magnum, which is used clinically in combination with symptomatology to determine if surgery is indicated in these patients, does not correlate with any feature of the CBF waveform. The changes in rCBF with CM may require further investigation since our measurements were performed at the frontal hemisphere but CM impacts the posterior fossa. There may be focal changes in the microvasculature blood flow and ICC/ICP that we are not capturing with this study. Interestingly, we also do not see a correlation between the pulsatile features and other systemic factors, such as MAP and BMI in CM patients. We see some correlation between each of the waveform features studied and HR in CM patients; however, we do not see this strong correlation between HR and the pulsatile waveform features in healthy controls (Fig. S3 in the Supplementary Material). Further work should be done to investigate how systemic factors impact these waveform features, and how this defers for various pathologies.

To our knowledge, this is the first study that investigates how the presence of a CM, a variety of physiological factors, and posture changes impact CBF morphology. Others have shown changes in baseline CBF with posture changes using DCS; they found that blood flow was higher in the supine position compared to the upright position, which is in agreement with our results in Fig. 5(a).^1^^,^^49^ However, here we also look at how these postures change the morphology of pulsatile rCBF waveforms.

In this work, we show how ICC impacts rCBF waveform morphology. We also introduced a method for peak identification that is more sensitive to subtle peaks compared to those previously used.^5^^,^^6^ This may further improve the ability to use morphological features of these pulsatile CBF waveforms as biomarkers.

## 5 Limitations

There are several limitations with this study. First, the SD distance we used to measure CBF was 2 cm and measurements may have significant scalp blood flow contamination. Although several other studies have used similar SD distances in adult populations,^50^^–^^52^ there is evidence that a longer SD or a combination of long and short SD distances may be more appropriate for adults because DCS is sensitive to extracerebral tissue contaminations.^53^^–^^55^ However, our pulsatile blood flow waveforms show three distinct peaks in the blood flow, which is indicative of good sensitivity to cerebral blood flow and has been shown by others in our lab to represent cerebral microvascular blood flow.^50^ Further, we applied external pressure to the probe by securing it with a tight head band. Previous work has shown that applying external pressure can help remove the scalp effects.^56^ Along with this, there were some instances where the Gaussian peak fitting approach did not lead to perfect peak finding. The performance of this approach was favorable to other methods, such as built-in peak finding algorithms; however, further work can be done to optimize this more and reduce error rates. Additionally, as mentioned, we are using the presence of a CM and older age as markers of impaired ICC; however, there are many variabilities between each of the individual participants that are not considered. For example, in this study, the race and sex distribution are different between the CM and healthy controls. Urner et al.^57^ published the work showing that males have a higher PI and a lower AUC compared to females in healthy subjects between the ages of 23 to 27 years old. In Fig. S4 in the Supplementary Material, we look at how sex may impact the pulsatile features in our data and find that in healthy controls there were no significant differences between males and females. Despite seeing similar trends to those reported by Urner et al., the differences between sex was not statistically significant in our data; this may be accounted for by the larger distribution of ages here.^57^ Additionally, CM mostly impacts the posterior fossa, but we are measuring blood flow in the frontal lobe. Previous studies have indicated that CM impacts global ICC, but perhaps we would have seen a more significant impact postoperatively if we measured CBF in the microvasculature of another region of the brain. There may also be individual differences in baseline amounts of ICC depending on lifestyle modifications, such as diet and exercise, which are not accounted for by the current study. Finally, there were differences in how certain postures were performed due to participant-specific factors like pain, flexibility, and body composition. Thus a larger cohort of CM patients may be warranted in future studies to minimize the effect of these potentially confounding variables.

## 6 Future Work

In this study, we only had a single timepoint of BP measurements taken with an automatic BP cuff and we did not have NIRS. Future studies should incorporate these to provide an external source of cerebrovascular compliance measurements as described by Baker et al.^11^ and Yang et al.^50^ There have also been methods introduced to alter ICC directly using saline infusions.^58^ Real-time DCS recordings during these could quantify how much extravascular compliance impacts CBF waveforms and could be used to develop models that decouple the impact of intra- and extravascular compliance. Adding simultaneous TCD could be considered, however, DCS has been validated against TCD in several other works. In addition, TCD is measuring blood flow in a different vascular bed with distinct properties and thus does not serve as a direct comparison. Beyond this, CM patients have relatively normal baseline ICP levels. Future studies could focus on other pathologies, such as idiopathic intracranial hypertension or hydrocephalus, where both ICP and ICC are impacted. Another option would be to place the probe intraoperatively, before dural opening in CM patients, and compare this to after the dura has been modified with duraplasty. Given that past studies have shown the potential for pulsatile DCS waveforms to predict ICP, morphological features may change more drastically in these cases. Finally, to our knowledge, this is the first study that describes age-related changes in pulsatile CBF waveforms in the DCS field. Although the primary goal of this work was to evaluate whether pulsatile CBF waveforms change with ICC, specifically changes in extravascular compliance, the age-related changes described suggest that DCS may be useful in other applications, such as a biomarker for small vessel disease and vascular dementia.^38^ It is also useful to know that the pulsatile waveforms change with postural changes since this may guide study design.

## 7 Conclusion

We investigated the impacts of altered ICC on the pulsatile CBF waveforms. Our results indicate that age most significantly impacted the morphology of the pulsatile waveforms in the cerebral microvasculature. There are some differences present between CM patients and healthy controls, but this difference was smaller, suggesting that there may be greater influence of cerebrovascular compliance compared to extravascular compliance on the waveforms. Here we looked at three features of the pulsatile waveforms: AIx, PI, and AUC. We found that AIx was the feature most impacted by ICC. We also saw that the features changed significantly with changes in posture. As the use of DCS and pulsatile CBF waveforms increases, it becomes important to understand what physiological factors impact these waveforms and which features may be used as clinical biomarkers.

## Data Availability

Signal processing code and examples of unprocessed CBF data will be made available at 10.1184/R1/24952731. The full dataset can be made available upon request.
