# Supplementary material for: Pulsatile microvascular cerebral blood flow waveforms change with intracranial compliance and age
Source: Neurophotonics. 2024 Jan 20;11(1):015003. doi: 10.1117/1.NPh.11.1.015003 (PMC10799239; doi:10.1117/1.NPh.11.1.015003)
Supplement: Supplementary file 1 [file NPh_011_015003_SD001.pdf]

Supplementary Figures

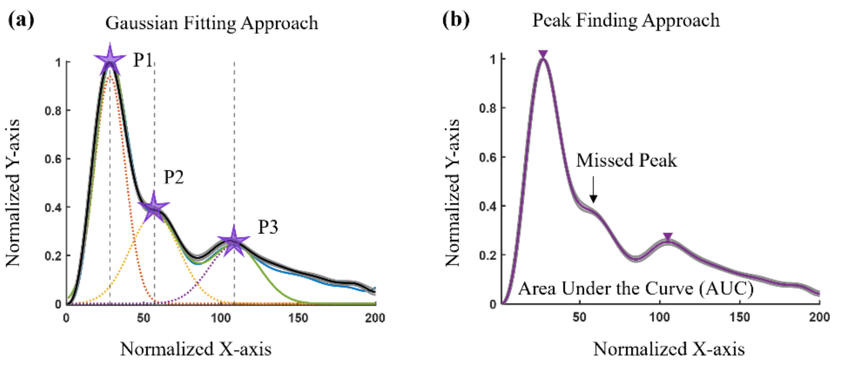

Supplemental Figure 1. Comparison of the (a) gaussian fitting approach with traditional peak finding approaches such as using (b) 'findpeaks' on Matlab .

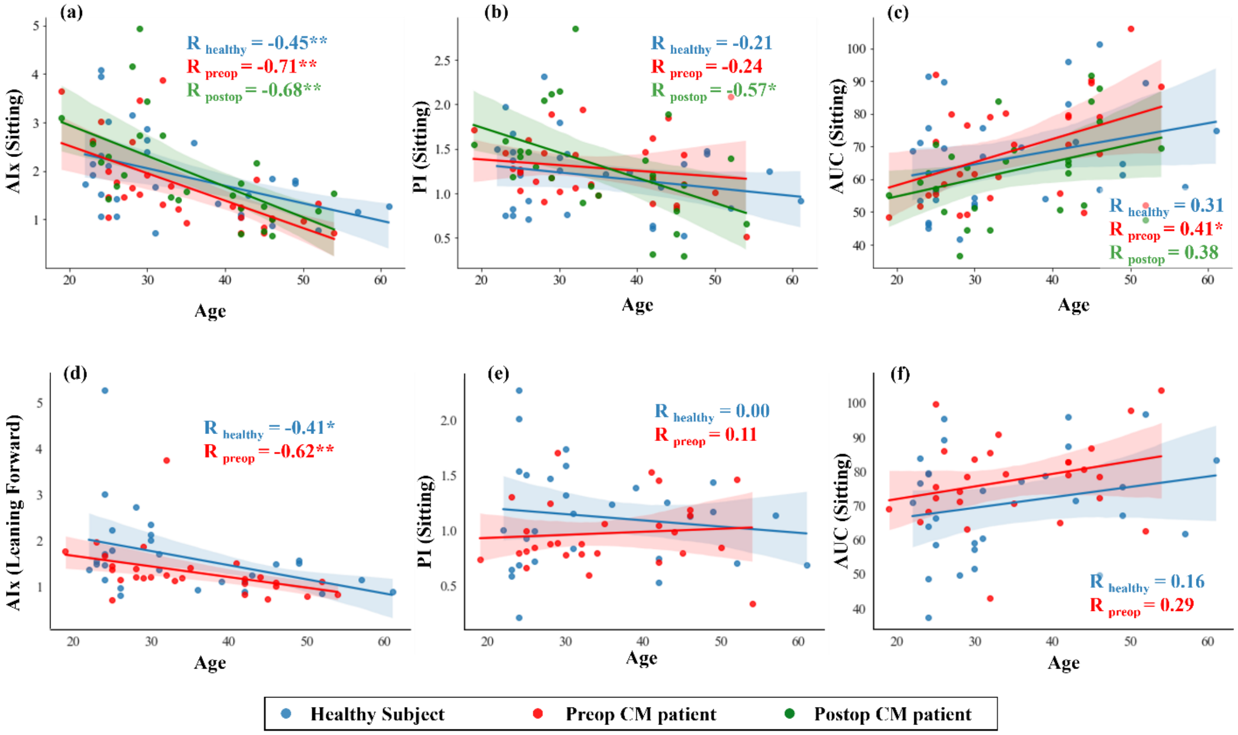

Supplemental Figure 2. Correlation of the pulsatile rCBF waveform features, (a & d) AIx, (b & e) PI, and (c & f) AUC, with age in the sitting position (a-c) and the leaning forward position (d-f). For the sitting position, data from preoperative and postoperative CM patients are shown, however, no data was collected from postoperative patients in this posture. Spearman correlation coefficients are displayed for each group, one star indicates a p<0.05 and two stars indicate a p<0.01.

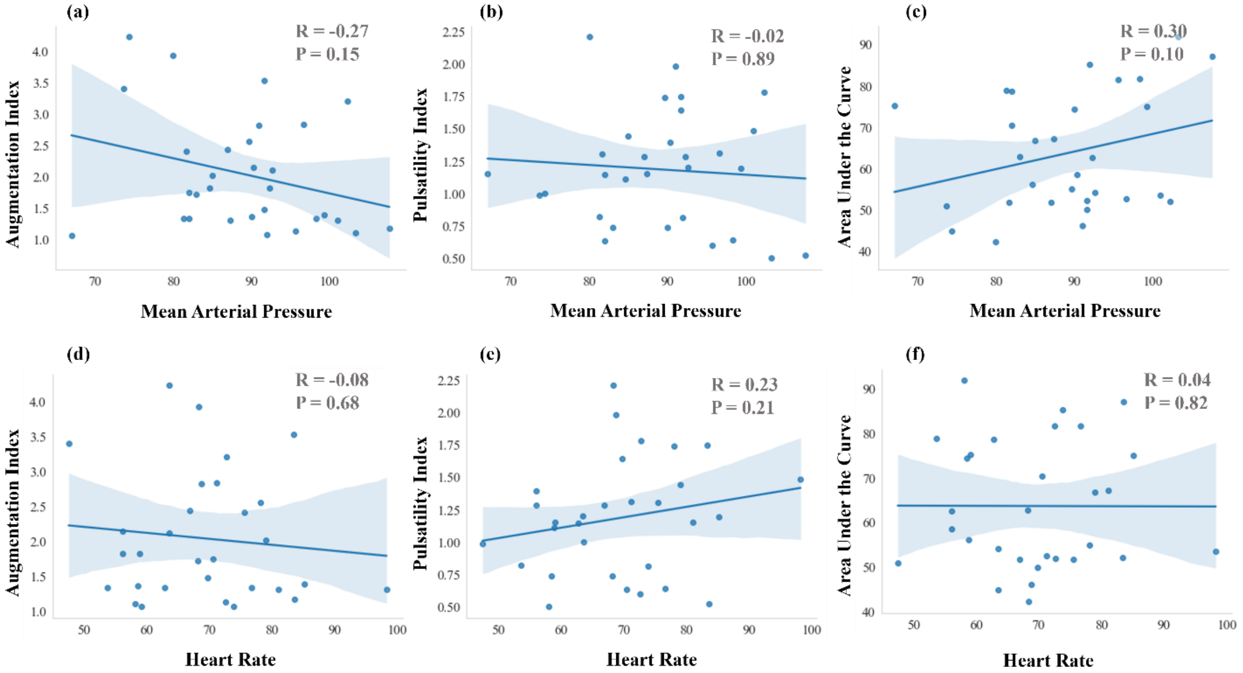

Supplemental Figure 3. Correlation of the pulsatile rCBF waveform features with MAP and HR in healthy controls while in the supine position. None of the rCBF features showed statistically significant correlation with MAP or HR.

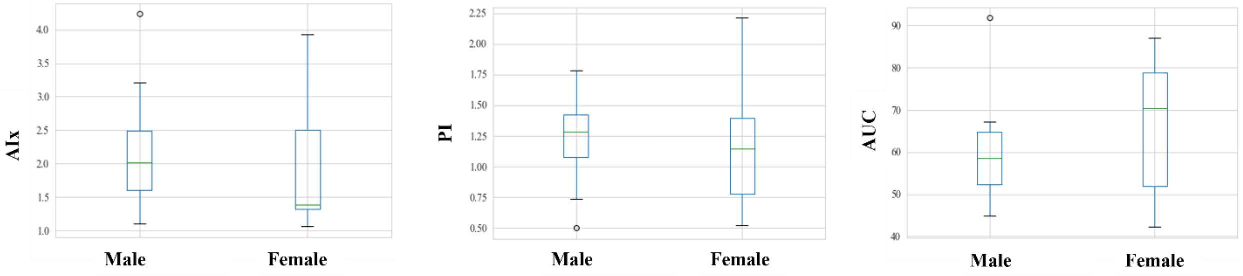

Supplemental Figure 4. Box plots showing the differences corresponding to sex in the pulsatile features of the rCBF waveforms measured in healthy controls (supine position). No significant differences were seen between males and females.

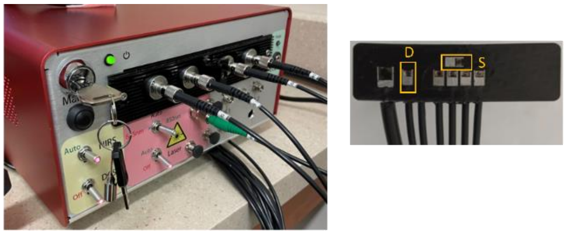

Supplemental Figure 5. Photo of the custom-built DCS system and probe.

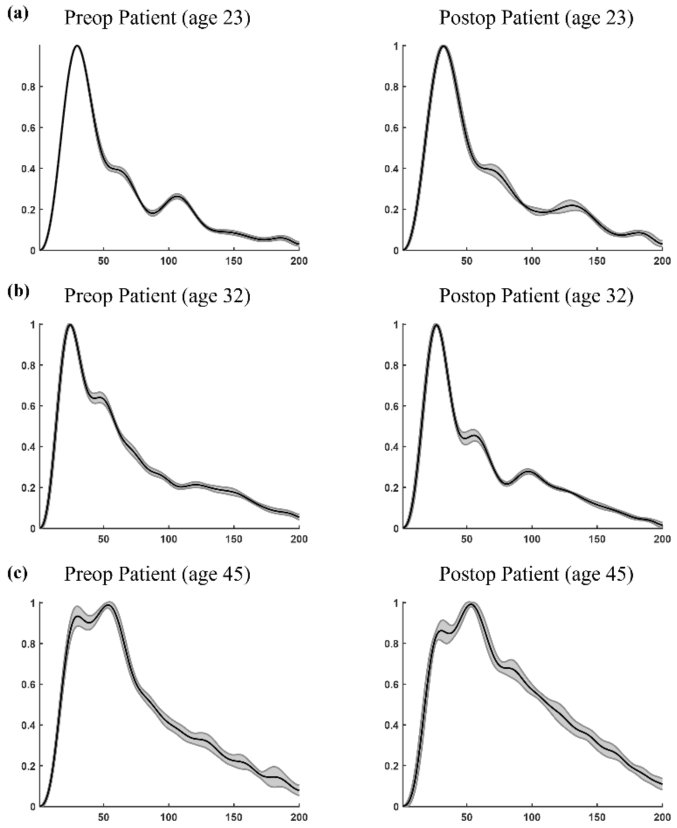

Supplemental Figure 6. Changes in rCBF waveforms from preop to postop in the supine position for three patients of different ages and sex: (a) 23/F, (b) 32/M, and (c) 45/F. The solid line is the average waveform while the patient is in the supine position and the gray error bar is the standard deviation.
